# The Role of Justice in Addressing the Social Determinants of Health

**DOI:** 10.1177/27551938251321973

**Published:** 2025-02-24

**Authors:** Sara Gilboe, Liz Curran

**Affiliations:** 1 Nottingham Law School, 6122 Nottingham Trent University, Nottingham, UK

**Keywords:** reducing inequality, justice and strong institutions, social determinants of health, health justice partnerships, justice, social justice, empowerment, impact, social change, evaluation of HJPs, access to justice, accountability

## Abstract

This article explores social determinants of health (SDH) in the global context and their connection to justice and human rights. Critiquing prevailing top-down approaches, it demonstrates that policy responses can be divorced from local factors necessary for addressing SDH. The authors examine health justice partnerships (HJPs) and how interdisciplinary collaboration between health and legal services can address inequality and health disparities. This discussion is timely given the Hague Declaration on Equal Access to Justice for All by 2030 formulates five pillars of people-centered justice. From the 2023 “Plan of Action” comes a “Joint Statement and Call to Action on the Rule of Law and People-Centered Justice: Renewing a Core Pillar of Democracy,” creating alignment between SDH and justice approaches for coordinated action. This article demonstrates how HJPs offer a unique avenue for driving change at the community level, advocating for systemic transformations to address poverty, inequality, and injustice. By driving change from local to international levels, multiple voices provide lenses for problem solving. Using American philosopher Carol C. Gould's theory of human rights, the authors explore how joint-disciplinary perspectives and moving beyond paternalistic intervention through integrating justice, human rights, and democracy can respond to drive outcomes in SDHs.

Worldwide, health inequalities persist mainly due to the inequitable distribution of social determinants of health (SDH). Despite efforts to improve interventions, low-income and vulnerable populations bear the brunt of unsuccessful attempts at targeting health inequalities. Reducing inequalities and promoting health equity have been categorized as a moral imperative.^1^ Calls to mitigate health disparities by interventions targeting well-being are increasing.^2^ Interventions on an international scale often lack accountability mechanisms and have limited success. Legal protections at a grassroots level might offer possibilities for bottom-up informing of international and national responsiveness. A new commitment and plan of action from the United Nations might see recognition of bottom-up approaches in justice linked to SDH, and people-centered justice finally being acknowledged.

The Summit for Democracy in 2023 with a “Joint Statement and Call to Action on the Rule of Law and People-Centered Justice: Renewing a Core Pillar of Democracy,” honors the 2019 Hague Declaration on Equal Access to Justice for All by 2030 (henceforth the Hague Declaration^3–5^). This declaration finally acknowledges unresolved legal problems linked to negative impacts on the health, income, and productivity of individuals and communities. It provides five pillars—to paraphrase, they include putting people and their legal needs at the center of justice systems; working to solve justice problems; improving justice journeys; using justice for prevention and to promote reconciliation; and empowering people to access services and opportunities.^3^ These international documents reinforce the approach of this article regarding the imperative for using justice in driving SDH outcomes.

The role of law and its legal support can impact health determinants in key ways: playing a normative role, challenging inequality in social conditions, offering alternative pathways through negotiating and brokering human rights adherence, and acting as a mechanism for societal structure development and policy and regulatory reform.^6^ Studies show that nine out of ten doctors working in low-income communities see the need for addressing social factors to influence patient health and the role that law can play.^1^ Increasing the use of law in addressing the impacts of SDH also increases accountability by ensuring lawfulness, increasing transparency, and providing advocacy for vulnerable populations. Law provides a voice for such populations when decisions affecting their lives are made by entities with power through raising awareness of frameworks and obligations.

This article explores the research question of how human rights law and justice approaches can increase accountability and create successful bottom-up initiatives. The authors discuss the model of health-justice partnerships (HJPs), where legal services work alongside health services. HJPs have the potential to shape and inform higher-level interventions to ensure relevance, responsiveness, and better adaptation than current models. At a local level, justice and health services innovate, playing a role in guiding future policies and addressing SDH to target justice and poor SDH outcomes.

The theory offered by Gould is that democracy can render some minority groups invisible as they lack resources, knowledge and capability.^7^ Gould suggests achieving human rights and justice action requires going beyond protecting basic legal rights of individuals against others or against the state.^8^

Gould asks what should be done when social and economic conditions limit self- development and participation. She notes even in democracies, some interests control the conditions needed by others for their self-development or “equal agency.” For example, resources in the power of nation states or laws that may operate unfairly can drive inequality.

Further, Gould argues that achieving human rights and justice in democracy requires enabling “equal positive freedom.” For this, humans must have access to the basic conditions they require to enact positive freedom for their self-development and to take part in common activities with others. She argues that self-transforming activity requires not only the making of choices but also the availability of the means or access to the social, political, and economic conditions necessary for making these choices effective.^8^

Accordingly, achieving a broader sense of justice requires rights to democratic participation, and accomplishes the following: forms of care and recognition across social spheres and borders; gender equality and overcoming oppressive social relations; “power-with” rather than “power-over” others; and the conditions for effective dialogue and deliberation.^7^

Operationalizing Gould's theoretical framework—especially with the endorsement from the U.N. people-centered justice^3,5^—can occur through legal assistance services (namely publicly funded legal aid and nonprofit free supports, such as law centers) working side-by-side with health services. Building justice options into health service provider repertoires can improve SDH by using the law to advise, protect or encourage adherence to human rights laws which would not otherwise be considered as options. For example, this can be achieved through raising awareness of legal remedies, enhancing advocacy, legal literacy, and capacity for earlier intervention through problem identification and increasing options which are often overlooked in the health repertoire.^9,10^ This approach is already making a difference to SDH outcomes. It is important to note, though, that awareness of legal remedies is not confined to court or adversarial options.^11^

Such legal support in health settings influences upstream and downstream service levels. It also enhances compliance and accountability through supporting grassroots initiatives guided by the community with frontline service delivery support blending justice with health equity. Promoting expanded avenues and broadening options to include justice solutions (beyond health and social considerations) increase responsiveness and accountability. These actions create the conditions for effective dialogue and deliberation that Gould argues are key to creating the availability of the means, or access to the social, political, and economic conditions necessary for making these choices effective.

The SDHs most relevant to the role legal support can play are income and social protection; employment and job security; working conditions; housing and environment; social inclusion and non-discrimination. However, all SDH can have a legal element as they all sit within legal frameworks. It is now increasingly necessary to consider the role of building legal capability of the community, as the people in health service delivery and policy are also critical players in the efforts needed to improve SDH. This article provides brief context on how current international governance—for example, the World Health Organization (WHO)—can play a role in expanding SDH to integrate social, legal, and health considerations. It argues that the role of law and options it provides in protecting, adhering to and honoring human rights, and enabling positive SDH outcomes has been overlooked for too long. The problems with international governance are that existing approaches have been limited in success as they tend to be abstract, top-down efforts often unlinked to lived experience of poverty, disadvantage and minoritization.

Discussion surrounding the effectiveness of attempts to decrease health inequalities globally highlights two key insights: health and SDH prioritization, and the undeniable link between health and human rights.^12^ Interventions targeting inequalities, SDH, and human rights law using human rights frameworks and legal services support can transform opportunities at an intergovernmental and national level, making headway in improving SDH outcomes. Justice and health services can then facilitate meaningful change, offering unique insights through their combined lens.

## Definitions

This article adopts the WHO's constitutional definition of *health* as “a state of complete physical, mental and social well-being and not merely the absence of disease or infirmity” (^13^, p. 1). While historical debates on health definitions exist, they are beyond the scope of this article. Any definition encompassing broader health implications is considered acceptable; however, the WHO definition aligns with global governance and leadership. It characterizes SDH as *nonmedical factors influencing health outcomes*, encompassing conditions in which individuals are born, grow, work, live, and age, along with broader forces shaping daily life.^14^ This definition contrasts with the term *underlying determinants* used in European and intergovernmental organization documents, where the term's use may lead to varied interpretations.^15^ Despite the WHO's definition, SDH often do not take precedence in responses operationalized by international bodies.

While each country can set its own list of SDH priorities, generally they are as follows^14^:

1. Income and social protection
2. Education
3. Unemployment and job insecurity
4. Working life conditions
5. Food insecurity
6. Housing, basic amenities, and the environment
7. Early childhood development
8. Social inclusion and non-discrimination
9. Structural conflict
10. Access to affordable health services of decent quality

Noticeable is the absence of equality before the law as a key underpinning of the rule of law being a precondition to democratic inclusion. The authors argue that this deficit, and the linking of SDH to the five pillars of the Hague Declaration, provide renewed opportunity to assist in identifying and addressing systemic inequalities intricately tied to limiting SDH. The failures in addressing systemic causes of inequality contribute to disparate health outcomes. By involving dialogues that include justice, often such systemic causes of inequality can be challenged and addressed, with poor or maladministration reduced by using justice mechanisms to enable accountability and lawfulness. Inequality occurs through variations in access to resources, opportunities, and social advantages. These inequities affect lower-income and vulnerable groups due to factors such as race, socioeconomic status, or religion and are often beyond individual control.^16^

Traditionally, the link between health and equal opportunity has centered on universal access to modern healthcare facilities—hospitals, clinics, and first aid resources.^6^ However, overemphasizing individual responsibility and health worker accountability overlooks the crucial roles of legislation and government in addressing SDH, perpetuating the misconception that individuals and health care workers bear sole accountability for a nation's health. Health and social issues are governed by laws that are often not understood, known or identified. This means that protections are ignored by authorities vested with important decision making. Such decision making affects lives, such as the rights to housing, income support, special needs assistance, and so on. For too long, the legal frameworks and adherence to human rights and their role in SDH have been underestimated.

Different governments adopt varied lists of SDHs. For example, the United Kingdom emphasizes a focus on child development and educational attainment, employment, living standards, and sustainable communities.^17^ The United Kingdom provides a determinant of health resource kit, with updated information on SDH within the country to reduce health inequalities. Although primarily intended for the public health system, it is publicly available and aims to provide determinant indicators, discussing the relationship between SDH and health outcomes.^18^ While countries may change the way they present SDH, there is clear recognition of their impact and importance.

## Context

### Current Limitations at an International Level

Health initiatives are typically overseen by bodies lacking legally binding powers. The WHO and its (optional) member states imbued social influence on health globally post-World War II through the constitution, formally defining health and SDH.^13^

The WHO, under the United Nations, is an agency that oversees international public health, collaborating globally for health promotion, safety, and universal health care. SDH gained prominence in WHO missions after the constitutional health definition,^13^ which explicitly includes legal connotations, such as the concept of duty-bearing (governments) and policies (adequate provisions and measures) that suggest inherent entanglement between health as a human right and law.

Optional participation enables member states to avoid committing to directives, leading to a lack of clear responsibility and expectations and is, therefore, a shortcoming of the organization. This disconnect undermines attempts at establishing accountability mechanisms and hinders consistent implementation of guidelines, compromising the organization's efficacy in addressing SDH.^19^ Despite the WHO's authority to ensure accountability via the World Health Assembly, as granted in its constitution, this power has been sparingly exercised.^13,20^ Instead, leading organizations in health have been reserved in their willingness to dive into legal realms.^21^

With the institution of the Hague Declaration's five pillars, participation levels might change. Previously, legislation, and legal tools deemed necessary for some health interventions were limited—as seen in the Framework Convention on Tobacco Control, where the WHO commented that “Legislation is key to effective tobacco control. It institutionalizes and makes binding a country's commitment. … and regulates private and public conduct in ways that voluntary measures cannot”.^22^ This legislative acknowledgment is a sign of potential in the context of how infrequently legislative powers have been used: only three times in seven decades.^21^

Amid gaps in the WHO's accountability measures, a phenomenon known as *inactivism* arose, with organizations hoping to pave the way for more concrete health and human rights directives entering the realm of providing services to international organizations.^23,24^ Consequentially, new challenges imposed upon global health leaders, like the WHO, stem from the division of the international community and the expanding architecture. This inactivism may now have shifted with the 2023 U.N. “Plan of Action”.^3,5^ Encouragingly, the Organisation for Economic Co-operation and Development (2023) has also supported the conception of people-centered justice. The United National Development Program has also provided guidance consistent with the arguments and the role of HJP asserted in this article. With this explicit acknowledgement of a role for access to justice at international level and its linking to attainment of SDGs, this article offers some practical methods to integrate people-centered justice movements by linking them to SDH and a role for new partnerships to expand their repertoire of responses to include justice.

Funding for initiatives now goes through various organizations, particularly donors, creating differences in task prioritization from agency to agency. Divided and disjointed initiatives not only disperse focus from goals set by the WHO but also goals with different political motivations can be prioritized. Uncoordinated interventions lead to financial loss and reduced impact due to duplicated efforts,^25^ emphasizing that stronger global leadership and coordinated local responses to address SDH effectively are required.

### Problems with Narrow Views of Health at a Global Level

Many countries aim to provide health indicators that relate to quality of life, which historically has been tracked economically through measurements such as gross domestic product. Such narrowing of tracking to only economic concerns omits social and human factors that better describe satisfaction and healthy living.^26^ Intersectional approaches to SDH are a requirement given the complex relationship between determinants and socially constructed systems, such as government and legal structures. Living an optimally healthy life for many individuals requires interventions that prioritize communities and health justice, with equity allowing for adequate resource allocation to address disparities. Often legal avenues can address poorly administered interventions.

### Theoretical Considerations

The 2030 United Nations Sustainable Development Agenda, known as the Sustainable Development Goals (SDGs), adopted by all U.N. member states in 2015, seeks global collaboration to address issues like poverty and deprivations. The agenda emphasizes strategies targeting health, education, and inequalities, aligning with SDH discourse, though it does not explicitly state this focus.^27^ While all goals have some ties to health, the following goals have immeasurably strong connections to SDH and justice:

Goal 1: No poverty
Goal 3: Good health and well-being
Goal 5: Gender equality
Goal 10: Reduced inequalities
Goal 16.3: The role of the rule of law at national and international levels and ensuring equal access to justice for all.

In 2005, the WHO established the Commission on SDH to provide support in targeting the social factors and determinants that impact health.^28,29^ Concerns emerge in reversed progress on infrastructure development, social, economic, and political inclusion, and effective institutions for peace and justice. There is insufficient data for targets like inclusive decision making, inclusive governance, elimination of discrimination, and equal economic rights.^30^

Using inputs from historically subordinated groups, as opposed to elite groups, represents a bottom-up rather than a technocratic, top-down approach. The authors argue that this disconnection from lived experiences leads to a bluntness in policy responses, rendering them ineffective in practice. Given powerlessness and lack of resources of these subordinated groups, there is a struggle to ensure accountability.^31,32^ The legal and governance systems are traditionally reluctant or unable to address issues affecting people facing adverse SDH impacts. The European Commission, representing the U.N. member states in Europe, has responded by adopting a holistic approach, placing SDG at the core of policymaking.^33^ Through policy mapping, the European Union documents policies and legal acts, addressing each SDG aim to centralize and uphold the SDGs importance.^34^ As noted, also at work are the five pillars connected to access to justice in the Hague Declaration.^4^ These pillars could be used to move beyond siloed approaches to find the role for justice in achieving the SDGs by improving access to justice in communities through a stronger linking and awareness raising of the role of justice in improving SDH outcomes. These documents show in principle effort toward increasing accountability within European countries and internationally, despite practical progress being limited. Many previous initiatives were responses to the COVID-19 pandemic, such as the newly established Health Emergency Preparedness and Response Authority, established as a commission service.^35^ The United Kingdom now faces new challenges in contributing to health-related goals due to Brexit, austerity, and cost of living increases.

In the SDH context, the global community acknowledges that SDH lie beyond individual control. Control is by social structures and institutions such as governments, and other governing bodies.^36^ Political systems, legislative systems (often neglected until recent literature), social policies, and economic policies are all areas where SDH can be impacted. Data suggest these factors influence over half of all health outcomes, far exceeding the influence of the health system and health workers alone.^14^ General health gains have disproportionately benefited those not necessarily needing intervention, leaving lower and middle classes behind, perpetuating associated poor health experiences.^37^

Poverty, an SDH, impacts other SDH. The United Kingdom is currently facing its highest poverty rate this century. Experiencing poverty makes individuals and communities more likely to experience poor nutrition, chronic disease, mental health problems, substance abuse, and results in more dangerous behaviors.^38^ Poverty statistics for the United Kingdom suggest that more than one in five live in poverty—14.5 million people—and nearly one in three children—4.3 million individuals—live in poverty.^39^ Despite attempted intervention, government approaches implemented from the top are not protecting the health of those most vulnerable, to the point where individuals in careers providing health-related care are themselves suffering.^40^ Unsuccessful interventions bring concern regarding the soaring cost of living increasing negative experiences with SDH.^41^

Gould's framework holds relevance with potential to expand approaches to the SDH by addressing her third point through “power-with” rather than “power-over” others and is central for democratic participation. Gould's theory emphasizes that achieving human rights and justice necessitates setting objectives for the development of political, economic, and social institutions that facilitate positive freedom. Central is individuals having access to essential conditions that are required to exercise their positive freedom. Key is having the necessary means to effectively implement these decisions.^8^

Legal services can assist in effectively implementing these decisions by identifying problems that have a legal solution early on (where legal interventions might have a positive impact on SDH outcomes) and, if necessary, empowering others or acting on their behalf to challenge unlawful conduct and poor responsiveness, thus ensuring accountability. Legal services use human rights protections and activate legal remedies often not through courts or litigation (as is commonly misconceived), but through negotiating with decision makers about their obligations, collective action, policy work, and solution finding. Most community members do not have the funding to mount legal cases, and community legal services are often adept at using the law to remind authorities and health services of obligations.^8,42^ When health and justice services work together, their joint perspectives can identify different mechanisms that can be deployed to intervene in poor SDH outcomes, such as inhumane housing, inappropriate evictions, and rejection from income support, which laws require. Inroads can occur through legal advocacy, legal education, and support for health professionals and lawyers who negotiate improved outcomes by working with decision makers to remind them of their responsibilities at law and to suggest adherence to these responsibilities. This interdisciplinary approach broadens dialogues to include such legal standards, therefore improving awareness of obligations and forging better compliance with them. By doing so, it brings action on SDH into line with the fifth pillar of the Hague Declaration, namely, empowering people to access services and opportunities.

## Methods

### Addressing Problems with Narrow Views of Health at a Global Level: the Potential of Integrating a Justice Lens

In addition to using Gould's framework to answer the central research question, the authors examined academic databases, employing a range of keywords to ensure thorough coverage of relevant literature in mainly common law countries (such as Canada, United States, United Kingdom, Australia, and India). These included varying combinations of SDH, justice, HJP, multidisciplinary practices, evaluation, effective service delivery, movement lawyering, collaboration, effective partnerships, strategic approaches to problem solving, human rights, participatory democracy, deliberative democracy, responsiveness to SDH, community organizing, community development approaches, law reform effectiveness, HJP evaluation, impacts of HJPs, medical–legal partnerships, response justice, access to justice, and SDH outcomes. The results were narrowed by determining whether a source was relevant to the global contextualization of SDH and to the links to justice and human rights. The literature review encompassed both academic and grey literature sources, including reports from international bodies, government agencies, and community-based organizations. The aim was to broaden the results beyond scholarly sources identifying intersections with justice and human rights and effective means for responding. Expanding the search included examination of sources of information where lived experience at a local level, and barriers and breakthroughs in improving SDH outcomes, were captured in data. This analysis of the literature revealed the role of HJPs in a number of jurisdictions and their integration of justice and health collaborations as a mechanism for improving SDH and accordingly these partnerships are explored in this article.

After reviewing the literature, various theoretical frameworks were examined, particularly focusing on human rights-based approaches through a social justice lens, as advocated by Gould. Utilizing thematic analysis, the aim was to identify effective methods of addressing SDH and promoting justice. Specifically, analysis focused on understanding localized phenomena that integrate justice with health concerns by considering community experiences; identifying models that transcend traditional direct service delivery to address underlying systemic causes of human rights issues; exploring justice responses aimed at noncompliance by authorities leading to improved outcomes in SDH and justice; and investigating strategies focused on systemic collaboration with local communities to facilitate changes in policy and funding, thereby enhancing community-based outcomes. HJPs were identified as providing potential in westernized nations, and the models can also be explored in other countries.

The subsequent findings and discussion are organized by themes that encapsulate the multifaceted role of law in addressing SDH and advancing justice and human rights. The first theme explores the pivotal role of law in shaping SDH outcomes, while the second theme delves into how law and human rights can promote accountability. The third theme examines legal assistance services, while the fourth theme investigates the potential of HJPs. Through these themes, the authors explore the complex interplay between law, SDH, and human rights, offering strategies for advancing health equity and justice.

## Findings and Discussion

Health, as a fundamental right, necessitates addressing SDH and inequalities through plans, policies, and strategies. These disparities adversely affect specific communities, resulting in a significantly lower quality of life.^43^ The root cause of societal limitations and health barriers often lies in institutions and legal frameworks set by governing bodies or the United Nations—which currently favor the health privileged—and a failure to seriously consider local conditions, many of which can be systemic in nature, that cause and entrench inequality.^44^ Active advocacy and empowerment, including legal capability not only of community members but of the services that support them, is crucial to addressing the realities faced by vulnerable and poorer community segments.

Health services witness firsthand the barriers that cause poor SDH outcomes. By collaborating with legal support and expertise, advocacy can be enhanced. HJPs utilize local health and legal services, mobilizing communities to shape improvements and influence policy, funding, and reform directly. This approach challenges the perception that the health rights of vulnerable and poorer populations are merely part of processes, emphasizing the need for systemic reform and accountability and countering the predominant benefit to people who are already well-resourced and powerful.^43^

Using Gould's requirement for positive freedom and self-development, the authors found that HJPs present an opportunity to connect and empower communities, a connection that can generate action.

### Theme 1: The Role of Law in Social Determinants of Health Outcomes

Reducing health inequalities and targeting SDH while prioritizing equity reduces the burden on health systems and fosters supportive, healthy communities.^45^ Traditional views must shift for equity and health justice, with a new grounding for communities and greater efforts at bottom-up approaches that start with the most vulnerable.^46,47^ Currently, planning is often dominated by top-down elites and the powerful organizations that miss the implications for communities that lack knowledge of legal remedies and the resources to advocate for themselves when they face structural barriers (such as poor decision making by authorities who control entitlements to basic needs). Top-down planning can poorly understand the impact of poverty, insecurity, and other barriers.^36^ The use of law in a bottom-up manner to address SDH has the potential to incorporate wider experience from the vantage point of those experiencing poverty and other barriers (disability, trauma, poor mental health, minoritization). This broadening of conceptions of how SDH can be improved generates views on problem solving that are more robust as they include input from affected communities on why they experience poor outcomes and are informed by what conditions can make a difference in their lives. Expanding views of SDH enables closer connections to lived experiences of individuals and the services that support them at a local level. This valuable insight can be filtered up to organizations at a national and international level to avert the current vacuum. HJPs emerge as a potential mechanism to provide a bridge and enable critical insights to filter and challenge conceptions that are informed by privileged conceptions and a lack of understanding of the practicalities in how policies play out on the ground.^42^ The literature examined suggests their work and policy input was aimed at connecting the local with the national and the international by looking at compliance, noncompliance, and the reasons for linking these with a feedback loop at each level. HJPs are evaluating effective practice, producing reports, and scholarly articles and identifying the role and potential power of this work.

### Theme 2: The Role of Law and Human Rights-based Approaches for Accountability in Health

In U.K. human rights law, “public authorities” are obligated to consider and balance human rights in their responses and decision making, with accountability for explaining and demonstrating their efforts using appropriate resources. If shortcomings are identified, an independent body can mandate redress, including accepting responsibility, revising policies, implementing new projects or plans, and providing financial compensation.^48^ Revised policies, projects, plans, and development are crucial tools for holding accountable those responsible for health inequalities and neglecting SDH for vulnerable populations. Accountability mechanisms, linked to these actions, promote health equity and reduce disparities. Legal assistance services in certain jurisdictions contribute to translating rights into reality often by indicating such obligations to those in power who Gould would describe as exercising control over peoples’ SDH outcomes. This recalibrated action can assist in redressing power imbalances.

Human rights-based approaches enhance accountability beyond judicial pathways by providing guidance, building legal capability and confidence, and facilitating early issue resolution. The acceleration of the right to health is achieved through fostering enabling environments, forming partnerships, and strengthening accountability. Advocacy, informed by persuasive arguments and a strategic understanding of lived experience and using this knowledge to negotiate with authorities (in which legal service can play a key role) can render minority groups less invisible, in line with Gould's call for democratic interplay. When a local community-based justice service collaborates with a health service, this capability is further enhanced, ensuring a close connection to community needs. HJPs amplify the perspectives and experiences of those without a voice to move to the forefront. By actively participating in direct service delivery and collaborating for improved policy responses and accountability, HJPs ensure they cannot be easily ignored.^49^ Emerging human rights approaches integrate community lawyers with people-centered services, targeting health issues at their roots through SDH and inclusive community-level decision making.^50^ The emphasis on community inclusion in health interventions is driven by human rights approaches and law. These improvements highlight the effectiveness of bottom-up initiatives as beneficial SDH interventions.^51^

### Theme 3: The Role of Legal Assistance Services in Improving Social Determinants of Health and Human Rights

Grassroots responses such as HJPs that emerged in the literature we examined see justice agencies partnering with health and allied health agencies at a localized level. These responses were born of a burning need to address the SDH outcomes and revolving door of recurring problems that, with holistic interventions, could be averted. The desire to address a problem at the root of its cause has led, and can lead, to better regulation or responsiveness. An illustrative example of this is where moldy homes see countless admissions of children with asthma to a hospital. An HJP can work to resolve the poor housing conditions that lead to the children's asthma by negotiation with the local authority, joining health expertise and knowledge of housing laws, improving existing or building better housing for people experiencing poverty.

HJPs are, based on the evaluation and other reports examined from the literature, ensuring improved and earlier responses, combatting siloed, fragmented, and often unnavigable service systems. They respond holistically to the problems experienced by people in disadvantaged situations. They see connections between health, social, economic, and legal problems. HJPs recognize that problems are likely to be connected, multiple, and interlinked as well as cascading in times of crisis.^16^ Recent research also provides evidence that trust is key if people are to reach out and seek help, and if agencies across the health and legal divide are to work together effectively to address SDH.^52^

Interventions targeting SDH and reducing inequalities have consisted of bottom-up initiatives involving communities and vulnerable populations.^50^ Conversely, governments and legislative changes frequently occur in a top-down, bureaucratic, exclusionary manner, deterring people from seeking help. These changes ignore systemic causes and attempt to regulate behaviors rather than understand how to elicit change.^53^

Bottom-up law understands how law affects community relations and social interactions, unpicking complexity in people's lives.^54^ Homogenous responses fall short, requiring tailored solutions aligned with on-the-ground action research rooted in experience. This approach drives policy, funding, and decision making from the bottom-up, ensuring that an interconnected web of health and effective interventions intersects with the law, demanding greater accountability. Human rights-based community law approaches provide a solution amidst the challenges of navigating change. Community-based initiatives, development plans, and greater engagement challenge health behavior actions by incorporating the voices of families and vulnerable groups.^55^ Frequently, vulnerable communities face top-down systems focused on cost-saving measures that reduce service accessibility.^44^ Collaborations between law centers and grassroots organizations act as a bridge, advocating for accountability and connecting the vulnerable with necessary services. Human rights-based legal approaches show success in building professional capability among health professionals to recognize the law's role in improving SDH and enhancing community capacity to seek help through engagement and timely support, linking them with HJPs through engagement and timely place-based support.^52,56^

Nationally, health is typically addressed in isolation, overlooking local context, and often not joined up with the potential for solutions informed by human rights-based approaches informed by direct on-the ground experiences. Legal obligations and standards and empowering communities to uncover systemic inequities in SDH distribution are too often misunderstood or overlooked. Transparency prevents authorities from concealing failures, enhancing accountability, and enabling meaningful change to address underlying issues.

Many preconceived stereotypes associated with SDH are shaped by limited experiences, informed sometimes by unconscious bias. Such stereotypes include perceptions of laziness or lack of willingness to change one's health circumstances in deprived communities. Stereotyping results in the relationship between poor health and life opportunities impacted by health being seen as the responsibility of an individual rather than rooted in the systemic reasons that lead to poor outcomes, such as discrimination or poor administration.^6^ In everyday life many SDH have ties to laws and policies that can be either under or unfairly applied, such as housing, education, employment and income, or food insecurity.^57^ Unsurprisingly, law and health services are naturally progressing toward collaboration to overcome barriers of top-down approaches. If done well, collaboration will be the most effective method in the pursuit of health equity and mitigating the impact of SDH.^52,58–60^

### Theme 4: A Unique Avenue for Driving Change at the Community Level, but Also for Systemic Transformations

HJPs are bringing together the field of law, legal organizations, and health care organizations. HJPs are built on foundational understanding that SDH can dramatically impact one's health and manifest as legal needs,^58^ aiming to provide higher quality services and care by pursuing the key values of (*a*) the social, economic, and political context in which people live has a fundamental impact on health; (*b*) SDH often manifest in the form of legal needs^61^; and (*c*) lawyers have the special tools and skills to address these needs (for example, the art of persuasion, advocacy skills, and knowledge of legal remedies).^2^ Integrating legal workers into health care settings enhances attention on SDH and the role of law in health justice. Mutual respect, teamwork, and nonhierarchical ways of working are key in the more successful HJP enterprises.^52^ Collaboration between the health and legal fields also seeks to reach local officials and policymakers.^6^

### HJP Design and Function

HJPs can integrate health and justice, unifying primary health care, public health, and legal sectors to improve social conditions and associated health. HJPs extend the notion of “health in all policies,” which is fundamental in targeting SDH flows to have upstream effects on interdisciplinary settings at local, national and potentially international levels.^62^ In public health, upstream interventions focus more on SDH, contributing social factors to ill health as well as prevention. Downstream approaches are those that target individual behaviors and treatments using a medical and individualistic concept. In reducing the gap caused by health inequities, downstream approaches can often exacerbate social issues.^63^

For example, poverty and the impact it has on health is a complex relationship. Poverty can result in poor housing conditions, lack of health service access, job or food insecurity, unsafe environments, and discrimination. These issues impact both an individual's mental and physical health in negative ways, and all of these problems are considered sociolegal.^64^ Each issue^65,66^ has regulatory frameworks and laws that allow for the possibility of a reduction of these poor social experiences. In the United Kingdom, inaccessibility of services and lack of legal aid can be a barrier to addressing health issues.^67,68^ An array of needs has possible legal remedies, along with the associated types of unmet legal needs impacting health that HJPs can address.^58,69^

HJPs create change by providing four key elements:

1. legal assistance by training health providers,
2. transforming health and legal institutions and practice through education,
3. training within both relevant sectors and communities, fostering community trust and intervening before crisis through knowledge and capability, and
4. administrative changes by providing policies generated outside of clinical settings, highlighting existing policy failures, and identifying overlooked barriers for vulnerable communities.^58^

HJPs act as a logical bridge, assisting vulnerable people in accessing services to address SDH and realizing the impact of law on the population. Health inequities, like poverty, are more easily addressed under HJPs due to cross-sector communication and problem solving.^70^ Unmet legal needs related to SDH (for example, substandard housing or eviction) are critical determinants of health. HJPs involve lawyers working alongside health care teams for education, issue identification, and enforcing civil legal rights.^62^ Since health care is considered downstream, providers and policymakers can be unaware of upstream impacts or how to deal with them. For example, health officials may draft policy regarding unsafe levels of lead in housing, suggesting safety laws in existing housing developments, but in doing so are blind to the upstream implications of enforcement. Landlords might evict families to avoid dealing with enforcements or victims of lead poisoning. In this instance, HJPs would be able to aid in understanding the importance of lead safety laws, but also proper enforcement and awareness of housing and tenant law.^71^ This example also shows how the use of HJPs can more accurately identify overlooked legal and policy failures relating to enforcement accountability or application within existing law, which can be used to target community-based health inequalities and increase health justice.

### Further Reflection

A variety of studies examined in the literature documented benefits of an HJP approach, such as an increase in communities accessing health services. For example, in one study in the United States, after HJP housing interventions there was a 91 percent decrease in emergency visits for asthmatic adults. Outcomes of HJP intervention also showed reduced rates of abuse and neglect; intervention also improved prenatal health practices and pregnancy outcomes among the vulnerable and low-income populations studied.^62,72^ Data and research publications demonstrated benefits of HJPs in targeting SDH. Success stories relating to HJP use are also becoming more frequent and well documented.^52,73–80^

This important role for law in SDH outcomes remains underexplored and should come to the attention of international and national entities that seek to improve SDH. The authors found that the successful HJP models in the literature provided unique and effective methods for driving change at the community level and expanded opportunities for systemic transformation.^70^

The most effective HJP models in the literature examined were linked to their local communities and targeted specific disadvantaged cohorts. They employed nonjudgmental community lawyers who took a problem-solving approach and recognized the expertise of health teams—for example, in building legal staff awareness of issues about health, such as the impact on client behaviors of mental health and trauma. This meant that lawyers could adapt their approach to taking client instructions to be more effective and build trust to enable clients to feel safe in disclosing the information needed for the lawyers to better support a client. Similarly, the HJP personnel saw their role as not limited to engaging with their clients and local community through community-based practice but also providing input and active involvement in campaigns and policy reform that incorporated the health perspective. This dual perspective can make a case for reform more compelling.^42^ The effective models in the literature tended to have a dedicated board and governance structures that work together in partnership between health and justice in an interdisciplinary and reciprocal way.

A successful example occurs in Uganda, where health services reach many vulnerable populations^81^ in remote areas. Health programs are developed incorporating SDH and by examining what is socially just, and use this information as a lens to go beyond individual immediate need. The ability to advocate and work with local authorities on SDH can be more sustainable if social justice is also part of the discussions. This underpinning can forge approaches that not only address individual health needs but also look at measures that can reduce inequality in the whole community through using justice. There is a range of legal nongovernmental organizations in developing countries who could work closely with and alongside health providers and the community to leverage this sort of systemic change.

### Implications: The Preventative Capacity of HJPs

Despite being downstream, HJPs wield significant influence, promoting upstream policy change through joint advocacys with perspectives and intersecting expertise from justice and health fields. Their primary focus on individuals in people-centered justice extends to collecting and analyzing community data and developing diagnostic tools to identify policies that are failing communities they were meant to safeguard. For instance, tools might gather data on children developing asthma due to inadequate housing policy enforcement. Armed with such information, HJPs drive broader community health and legal interventions, ensuring that protective policies are enforced.^70^ The inclusion of a legal team advocating for health policy changes propels progress toward upstream transformation.^52^

HJPs uniquely identify systemic barriers in health and justice, treating and identifying upstream policy changes needed on larger scales.^52,82^ This approach harnesses law for systemic and structural changes, facilitating accountable actions. When engaged, this feature heightens preventative capacity by not only treating the symptoms of the problem but their cause.^58^ For example, treating recurring and severe life-threatening asthma and child illness in hospitals caused by ongoing poor housing conditions is expensive and ambulatory. Whereas, putting a compelling case to housing authorities based on medical evidence and redrafting regulation to make local authorities obliged to rectify or not provide poor housing averts the asthma in the first instance. This reduces morbidity and means health resources can be better deployed.^70^ Additionally, the legal sector is learning about its role in prevention and transformation due to its growth mindset that results from working closely with the health sector.^52^ Further engagement with structural change allows HJPs to function on a population level, although this area requires significant research to determine feasibility, scalability, and functionality.

## Limitations

Despite the number of benefits of utilizing HJPs, there are also limitations or, rather, concerns about their use and applicability. Some research showed a gap in empirical evidence regarding the effectiveness and capacity of HJPs in targeting the SDH over varying populations. In one instance, there was less evidence on the function of HJPs for individuals who experienced a broader array of health and legal concerns, some from social-related issues like the ones discussed mixed with some that may not have immediate or entire ties to social structure issues.^2^ This area demands further investigation, as many individuals experiencing health inequalities rarely suffer from only one, and unique or interrelated health–legal concerns are equally as common.^83^ The idea is that legal integration with the medical field needs to be far more expansive, but it is not easy. There is still distrust of lawyers among many in the health profession, stemming from the “win and lose” nature of the adversarial court system, experiences in medical negligence, and vigorous cross-examination.

Concerns also revolve around tracking success as HJPs grow. Success should not be superficially measured by referrals from clinical to social care (from health to legal). Referral data alone does not indicate if the agency took on the matter or if the intervention was effective. This reduces HJP success to a single criterion, neglecting documentation, tracking patient outcomes, service access, and health improvement. These concerns relate to political and governmental complexities, with underfunded and understaffed community-based initiatives worldwide.^70^ Health care providers are also often overloaded or understaffed.

Resistance to joining legal services by the health sector stems from negative experience in the legal system, especially aggressive cross-examination in medical negligence cases. As occurs in many HJPs, stereotypes need to be broken down, and restoring trust is crucial.^84^ Integrating legal representatives into health settings may face similar challenges. HJPs are not easy enterprises; they demand discussion, joint planning, and strong relationships between health and justice organizations at management, funding, and operational levels. Time needed in developing these relationships, breaking down stereotypes, identifying common values, and committing to holistic approaches often are not factored into funding and planning but are essential for successful HJPs.^52,85^

## Future Opportunities for HJPs

HJPs should be accessible in various safe spaces for communities, addressing historic difficulties faced by vulnerable populations in receiving quality care, such as indigenous populations in Canada in Australia. The second author is currently undertaking a three-year research impact evaluation of an HJP in rural regions in Australia with a focus on mental health in the First Nations community. Through on-the-ground work and research, community HJPs have shown the ability to create awareness of legal rights among unknowing individuals and to increase access to the right services and interventions. Ongoing supportive relationships between lawyers, health care providers, and patients enhance public trust and demonstrate that justice is possible, improving accountability in health responses to SDH locally, nationally, and globally.^86^

## Conclusion

The role of law in addressing SDH, reducing inequalities, and increasing accountability is evolving. While health is traditionally considered a field of its own, prioritizing health without considering other fields, like justice, is limiting progress on SDGs and SDH. The international movement, with the Hague Declaration and European Commission call for more holistic and people-centered responses, is a recent positive shift. The complexity and interconnectedness of SDH challenges traditional approaches. Despite policies and legislative changes, health inequalities persist from a lack of proper framing and accountability measures. Social factors that influence health are a human rights issue. Bottom-up human rights-based approaches, like well-designed HJPs, show how law working alongside health can create change, increase visibility of human rights protections, and elicit lived experience narratives leading to accountability. The authors conclude that linking SDH to a justice lens and endeavoring to actualize Gould's argument for democratization are possible for effective HJPs. Moreover, this collaborative approach can bring greater responsiveness and justice outcomes and reduce inequality and inequitable SDH distribution.
